# Particularities of preimmune reactivity in patients with pulmonary tuberculosis in combination with toxocariasis

**DOI:** 10.1186/1471-2334-13-S1-P80

**Published:** 2013-12-16

**Authors:** Valentina Smesnoi, Serghei Ghindă, Gheorghe Placintă

**Affiliations:** 1 Chiril Draganiuc Institute of Phthisiopneumology, Chişinău, Republic of Moldova; 2 Department of Infectious Diseases, Faculty for Continuing Medical Education, Nicolae Testemițanu State Medical and Pharmacy University, Chişinău, Republic of Moldova; 3 Toma Ciorbă Clinical Hospital for Infectious Diseases, Chişinău, Republic of Moldova

## Background

For many years it was considered that bronchial obstruction is caused by a bacterial or viral infection of the respiratory system and the pathogenesis is associated with the sensitization of a bronchial tree by pathogens or non-pathogenic entities which inhabit the respiratory passages. Symptomatic pulmonary disease characteristics are present in 65% of patients with visceral toxocariasis and catarrhal symptoms vary from mild to severe asthmatic.

## Methods

The study included 66 patients, out of which 20 had pulmonary tuberculosis, 20 had pulmonary tuberculosis and *Toxocara* coinfection, and 26 *Toxocara* infection. The levels of cytokines IL-4, anti-*Toxocara* IgG and IgE were determined by ELISA. To assess the functional activity of neutrophils the test nitro-blue tetrazolium (NBT) was used and we modified the method proposed by Park.

## Results

The study of cytokine profile (IL-4), Ig E levels, eosinophils and T lymphocytes proliferative activity in the reaction of blast transformation of lymphocytes demonstrated that for patients with tuberculosis and toxocariasis association the Th1/Th2 type immune response is characteristic. For patients with pulmonary tuberculosis the Th1 type immune response is characteristic, while for patients with toxocariasis is characteristic the Th2 type of immune response.

## Conclusion

In patients with toxocariasis without respiratory pathology we identified Th2 type of immune response to treatment and its dynamics. This could explain a more favorable prognosis for a respiratory pathology associated with toxocariasis.

